# Resembling Left Ventricular False Tendon in a Father and His Daughter

**DOI:** 10.1155/2018/9543098

**Published:** 2018-12-09

**Authors:** Abdullah Kaplan, Hacı Murat Gunes

**Affiliations:** Department of Cardiology, Istanbul Medipol University, Istanbul, Turkey

## Abstract

Left ventricular false tendons (LVFTs) are linear fibrous or fibromuscular bands stretching across left ventricular cavity. Although LVFTs have been associated with various heart pathologies and investigated embryologically and histologically, there is only one report in the literature connoting possible hereditary transmission of this entity. We reported a father and his daughter having similar types of LVFTs with regard to location and thickness. With this report, we will contribute in the literature in respect to potential genetic inherence of LVFTs.

## 1. Introduction

Left ventricular false tendons (LVFTs) are linear fibrous or fibromuscular bands that extend between ventricular septum and papillary muscle, left ventricular free wall, or apex [1, 2]. LVFTs were incidental findings at cadaver dissection in early days, but later technical improvement in the heart imaging such as echocardiography and magnetic resonance imaging let the percentage of LVFT visualization rise [2, 3]. LVFT prevalence in the literature spreads over a wide range, while in the earliest reports, it was 0.5%; current investigations have reported up to 78% [3].

Although there is no consensus on the implication of LVFTs in heart diseases, they have been associated with various clinical manifestations in otherwise structurally normal hearts such as functional murmur, preexcitation, idiopathic ventricular tachycardia, infective endocarditis, cavitary thrombi, regional myocardial hypertrophy, repolarization abnormalities, and genesis of J-waves [2–9].

Our knowledge about LVFT continuously grows. In order to understand clinical implication of LVFT, we need to evaluate this entity in all its aspects. The questions rose regarding its clinical implication let researches to extend histologic characteristic and embryologic development of LVFT. The lacking information regarding genetic transition of LVFT is the missing link of this chain. We offered an insight into genetic aspect of this entity by reporting these cases.

### 1.1. Case 1

**Figure 1:**
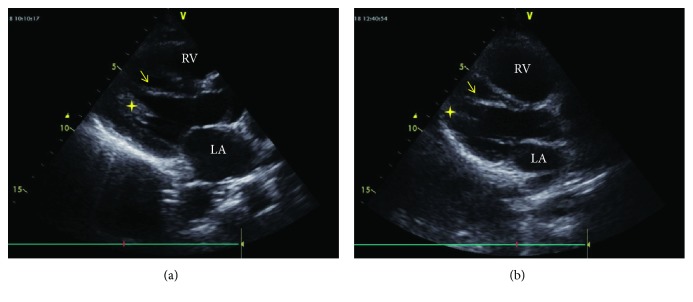
B-mode echocardiographic image from the father (a) and his daughter (b). (a) and (b) Modified parasternal long axis view by B-mode recording. The arrow indicates the false tendon stretching from the left ventricular apical free wall to the ventricular septum. The star indicates the papillary muscles. LA: left atrium; RV: right ventricle.

**Figure 2:**
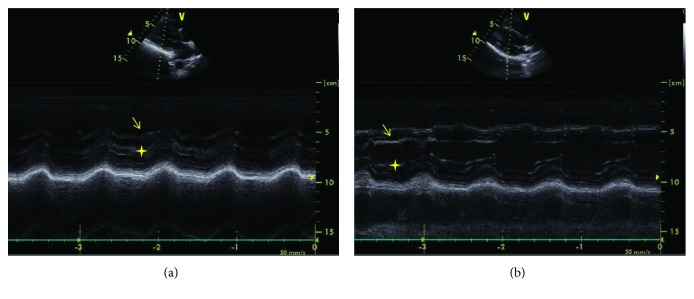
M-mode echocardiographic image from the father (a) and his daughter (b). (a) and (b) M-mode recording from the midsegment of the left ventricle. The arrow indicates the false tendon; The star indicates the papillary muscles.

**Figure 3:**
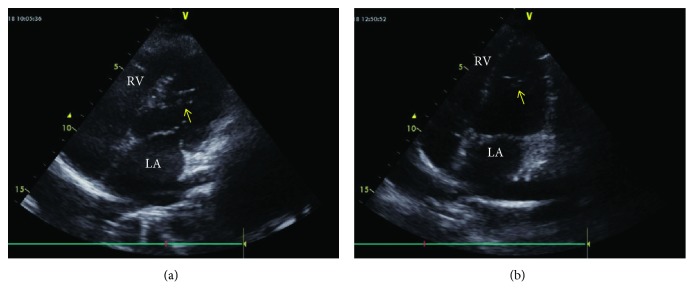
B-mode echocardiographic image from the father (a) and his daughter (b). (a) and (b) Modified apical four chamber view by B-mode recording. The arrow indicates the false tendon stretching from the left ventricular apical free wall to the ventricular septum. LA: left atrium; RV: right ventricle.

**Figure 4:**
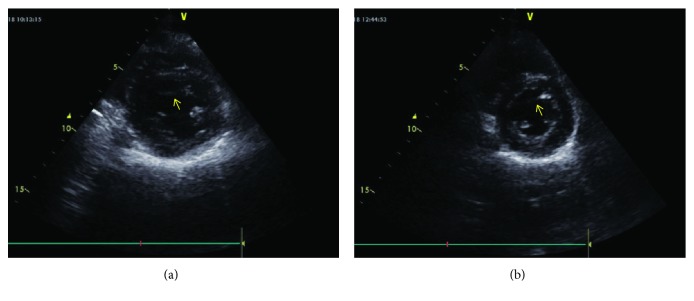
B-mode echocardiographic image from the father (a) and his daughter (b). (a) and (b) B-mode image from the short axis. The arrow indicates the false tendon stretching from the left ventricular free wall to the ventricular septum.

An 18-year-old previously healthy girl presented with complaint of palpitation. She has experienced palpitation 2 or 3 times a month for 3 years. She did not declare other triggering factors for palpitation with the exception of caffeine. She stated that her palpitation episode sometime lasts one hour, and it is rarely accompanied by dizziness. On physical examination, the followings were noted: normal S1 and S2 without added sounds (S3 or S4) and murmur on cardiac auscultation, blood pressure 118/76 mmHg, heart rate 78/min, and normal breathing sounds. A 12-lead electrocardiogram showed sinus rhythm with normal QRS morphology. Transthoracic echocardiography revealed a structurally normal heart with the exception of a broad false tendon within the left ventricle extending between apical lateral wall and basal septum (Figures 1(b)–4(b)). Ambulatory rhythm monitoring showed no isolated premature ventricular complexes, ventricular couplets, or runs during 24 hours. She was asymptomatic during the 24 hours of ambulatory rhythm monitoring. She was advised to obtain an electrocardiogram at the time of palpitation from the nearest medical center.

### 1.2. Case 2

The father of the girl in case 1, 52 years old, was admitted in our outpatient clinic due to a periodic examination of coronary artery disease. He had coronary bypass surgery history. At the time of admission, he was asymptomatic with regard to coronary artery disease. On physical examination, the followings were noted: normal S1 and S2 without added sounds (S3 or S4) and murmur on cardiac auscultation, blood pressure 132/82 mmHg, heart rate 72/min, normal breathing sounds, and no peripheral edema.

A 12-lead electrocardiogram revealed nonspecific ST changes in precordial leads. Transthoracic echocardiography showed normal systolic contraction in all left ventricular wall segments. There was no evidence of left ventricular cavity enlargement or hypertrophy according to the measurements suggested by chamber quantification guideline [10]. During the evaluation, a left ventricular false tendon extending between apical lateral wall and basal septum was noticed (Figures 1(a)–4(a)).

## 2. Discussion

Although there are several reports in the literature regarding embryologic development, histologic evaluation, and its clinical association of LVFT, to date, genetic aspect of LVFT has not been scrutinized thoroughly. Many years ago, genetic transmission of LVFT became the main topic of conversation upon a presentation of resembling LVFTs in a mother and her son in Russian literature [11]. Unlike the previous one, we reported LVFT in a father and his daughter with a similar point of attachment and thickness in the left ventricle. To our best knowledge, this is the second report to point out the potential genetic inheritance of LVFT in the literature.

Clinical implication of LVFTs is still not well understood although several reports have showed their association with some heart diseases [3]. In order to disclose a potential link between LVFT and heart disease, histologic feature and embryologic development of this entity have been studied. Although embryologic origin of LVFT has not been fully elucidated, they are thought to be derived from inner muscle layer of primitive heart [2, 12, 13]. Histologic analyses of LVFTs revealed that they can contain fibrous tissues, myocardial fibers, elastic fibers, and blood vessels [2, 14, 15]. LVFTs can be classified into three types based on their thickness although their functional differences remain unknown. Fibrous type is less than 1.4 mm in diameter, fibromuscular type is 1.5–2.4 mm, and muscular type is larger than 2.5 mm [2]. Our both cases had muscular type false tendon based on their thickness (Table 1).

LVFTs are classified into 5 types with varying percentages based on the points of attachment. Type 1 (66%) extends between posteromedial papillary muscle and ventricular septum, type 2 (12%) between anterolateral papillary muscle and posteromedial papillary muscle, type 3 (11%) between anterolateral papillary muscle and ventricular septum, type 4 (9%) between left ventricular free wall and ventricular septum, and type 5 (1%) between one left ventricular free wall and another left ventricular free wall [16]. Our both cases had type 4 LVFT based on the points of attachment where the tendons extended between apical lateral wall and basal septum (Figures 1–4).

Although there is no consensus on clinical importance of LVFTs [3], it might be important to visualize the false tendon by cardiac imaging in some cases. In those who undergo heart surgery or left ventricular catheter ablation, the presence of LVFT must be known by the operator to avoid damage to these tendons, in view of the possible presence of the conduction system and coronary artery branches in the tendons [2]. As well as, because LVFTs can be the origin of the infective endocarditis [8, 17] and cavitary thrombus [18, 19], clinician must be vigilant in those who have unknown source of fever or systemic embolism.

Several heart diseases and electrocardiographic abnormalities have been associated with LVFTs [2–4, 20–22]. However, in our both cases, we failed to show any heart pathology linked to LVFT as reported in the literature. Even though the girl presented with complaint of palpitation, ambulatory rhythm monitoring showed no arrhythmia during 24 hours. It is worth noting that she was asymptomatic during the recording.

By reporting these cases, we draw attention of clinicians on the potential genetic inheritance of LVFTs. Clinical studies are warranted to confirm the genetic inheritance and reveal the pattern of genetic transmission of LVFTs.

## Figures and Tables

**Table 1:** Maximum thickness of the left ventricular false tendon in both cases.

Subjects	PSLAX (mm)	SAX (mm)	A4C (mm)	Average (mm)
Father	8	3	9	6.6
Daughter	6	2	7	5

PSLAX: parasternal long axis; SAX: short axis; A4C: apical four chambers.
